# Ouabain Stimulates a Na^+^/K^+^-ATPase-Mediated SFK-Activated Signalling Pathway That Regulates Tight Junction Function in the Mouse Blastocyst

**DOI:** 10.1371/journal.pone.0023704

**Published:** 2011-08-25

**Authors:** Holly Giannatselis, Michele Calder, Andrew J. Watson

**Affiliations:** 1 Department of Obstetrics and Gynaecology, The University of Western Ontario, London, Ontario, Canada; 2 Department of Physiology and Pharmacology, The University of Western Ontario, London, Ontario, Canada; 3 Children's Health Research Institute, London, Ontario, Canada; 4 Lawson Health Research Institute, London, Ontario, Canada; University of Giessen Lung Center, Germany

## Abstract

The Na^+^/K^+^-ATPase plays a pivotal role during preimplantation development; it establishes a trans-epithelial ionic gradient that facilitates the formation of the fluid-filled blastocyst cavity, crucial for implantation and successful pregnancy. The Na^+^/K^+^-ATPase is also implicated in regulating tight junctions and cardiotonic steroid (CTS)-induced signal transduction via SRC. We investigated the expression of SRC family kinase (SFK) members, Src and Yes, during preimplantation development and determined whether SFK activity is required for blastocyst formation. Embryos were collected following super-ovulation of CD1 or MF1 female mice. RT-PCR was used to detect SFK mRNAs encoding *Src* and *Yes* throughout preimplantation development. SRC and YES protein were localized throughout preimplantation development. Treatment of mouse morulae with the SFK inhibitors PP2 and SU6656 for 18 hours resulted in a reversible blockade of progression to the blastocyst stage. Blastocysts treated with 10^−3^ M ouabain for 2 or 10 minutes and immediately immunostained for phosphorylation at SRC tyr418 displayed reduced phosphorylation while in contrast blastocysts treated with 10^−4^ M displayed increased tyr418 fluorescence. SFK inhibition increased and SFK activation reduced trophectoderm tight junction permeability in blastocysts. The results demonstrate that SFKs are expressed during preimplantation development and that SFK activity is required for blastocyst formation and is an important mediator of trophectoderm tight junction permeability.

## Introduction

Blastocyst formation is a prerequisite for the initiation of pregnancy, however, the majority of mammalian preimplantation embryos fail to complete this developmental interval and implant [1]–[5]. This restricted developmental success greatly reduces the efficiency of methods aimed at fostering both animal and human assisted reproduction. As such, there is a requirement to increase our understanding of the cellular and molecular mechanisms that control preimplantation development, and in particular, blastocyst formation [1]–[5]. In addition, preimplantation development encompasses the first cell differentiation events of development including the formation of the epithelial trophectoderm and the pluripotent inner cell mass [1]–[9]. Research directed at understanding the mechanisms that control trophectoderm differentiation, and thus blastocyst formation, also serves to provide fundamental insight into the mechanisms controlling epithelial cell differentiation throughout development and the mechanisms controlling acquisition of cell polarity [10]–[13].

Blastocyst formation is regulated by the combined actions of ion transporters, water channels, and intercellular junctions [1]–[3], [5]. We have hypothesized that blastocyst formation is regulated by the action of a polarized basolateral localized Na^+^/K^+^-ATPase that creates a trans-trophectodermal ion gradient [3], [14]–[25]. This facilitates water movement across the epithelium, in conjunction with aquaporin water channels, to form the blastoceolic fluid [16], [26], [27]. The blastocyst expands via the continued movement of this fluid across the epithelium, but this does not occur until a fully developed and functional tight junction complex between adjacent trophectoderm cells is formed [7], [14], [28]–[31]. Thus, blastocyst formation is regulated by the formation of this trophectoderm tight junctional seal. While research has uncovered the principal molecular constituents of the mechanism controlling blastocyst formation we know relatively little about the regulation of each individual component.

Ouabain is a cardiotonic steroid that is primarily known as a plant-derived chemical that specifically binds to the Na^+^/K^+^-ATPase to modulate the ion transport function of the pump [32]–[44]. Recent research has established that ouabain and other cardiotonic steroids are in fact a newly discovered group of endogenous steroid hormones that are produced primarily by the adrenal glands [32]–[44]. This discovery has directed research towards understanding the physiological roles of endogenous cardiotonic steroids in regulating Na^+^/K^+^-ATPase function [32]–[44].

In addition to regulating Na^+^/K^+^-ATPase ion transport, research applied primarily to cell lines has indicated that ouabain binding to the cell also regulates SRC pathway signalling [45]–[50]. These discoveries have indicated that ouabain binding to its Na^+^/K^+^-ATPase receptor regulates cellular function via activation of SRC and its downstream mechanisms [45]–[50]. We have hypothesized that this ouabain-mediated, SRC-activated pathway plays an important role in regulating preimplantation development by regulating trophectoderm tight junction function.

In this study we present evidence for the expression of *Src* family kinase members, Src and Yes, during preimplantation development. We establish concentrations of ouabain that both activate and inhibit SFK activation at the blastocyst stage. Furthermore, we demonstrate that SFK activity is necessary for blastocyst formation, and more specifically, regulates trophectoderm tight junction function. We therefore conclude that the developing blastocyst has the capacity to respond to ouabain by activating SFKs and that this process is an important mediator of tight junction function, and thus overall blastocyst formation.

## Results

### Detection of Src and Yes mRNAs during Mouse Preimplantation Development

**Figure 1:**
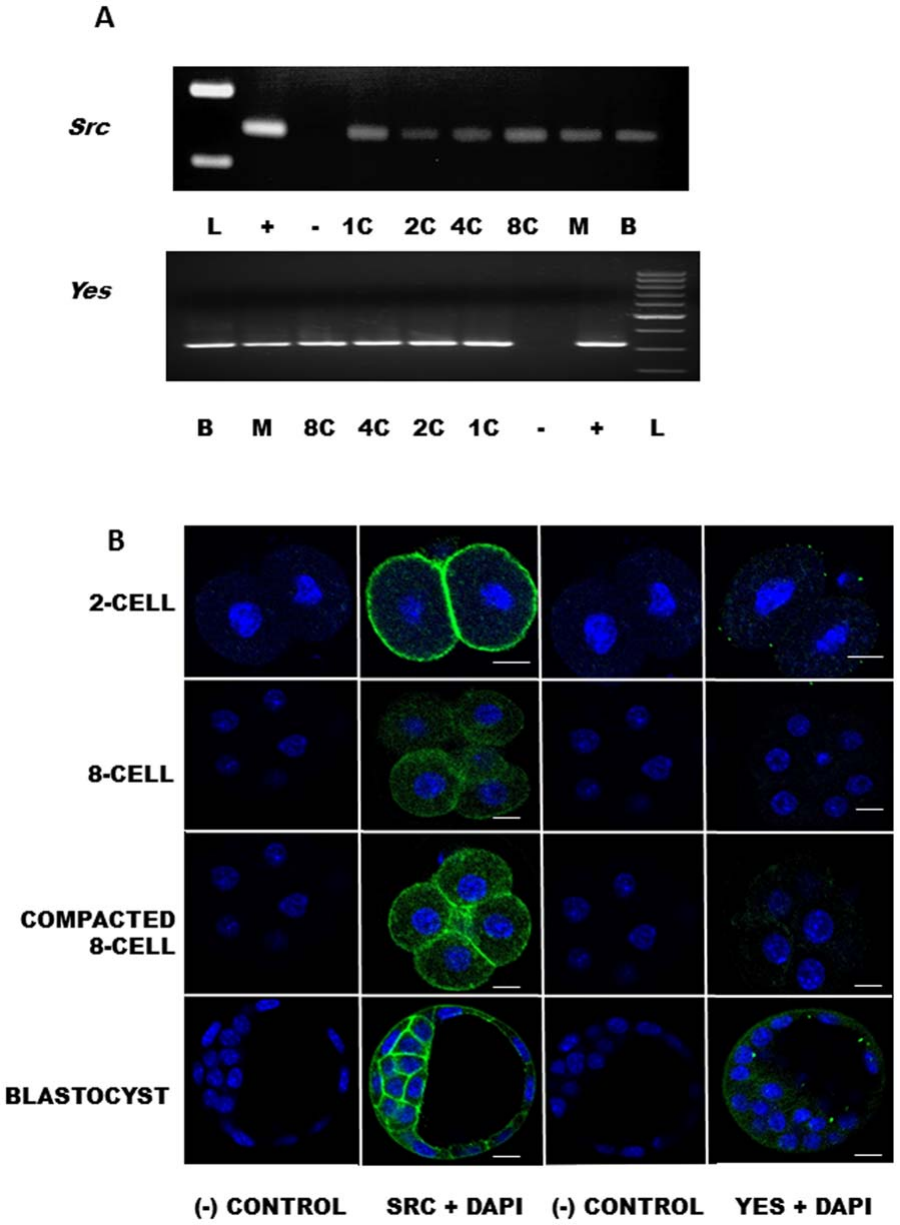
A,B. Detection of SFK (Src and Yes) mRNA and protein during Mouse Preimplantation Development. mRNAs encoding *Yes* and *Src* mRNAs were detected during preimplantation development (1C, 1-cell; 2C, 2-cell; 4C, 4-cell; 8C, 8-cell; M, morula; B, blastocyst; L, low mass ladder with 300 and 400 b.p. visible). Lung tissue RT positive controls (+) and no cDNA template negative controls (−) are shown. Representative of 3 independent experiments. In the 2-cell stage, SRC was localized to the cell cortex. SRC immunofluoresence diminished in the 8-cell embryo, but reappeared at areas of cellular contact in the compacted 8-cell embryo, as well as in all trophectoderm (TE) and inner cell mass (ICM) cells in blastocysts. At the 2-cell stage, YES immunofluorescence consisted of diffuse cytoplasmic staining. YES immunofluorescence diminished in the 8-cell embryo, but reappeared during compaction and was detectable in all cells, TE and ICM, of the blastocyst. Negative controls represent embryos incubated with FITC-conjugated secondary only (no primary antibody), in addition to DAPI. In all confocal micrographs, green indicated positive staining for proteins and blue indicates DAPI nuclear staining. Scale bars represent 10 µm.

*Src* and *Yes* mRNAs were consistently detected, using RT-PCR applied to three preimplantation development series (1-cell, 2-cell, 4-cell, 8-cell, morula and blastocysts) experimental replicates, throughout mouse preimplantation development (Figure 1A). The PCR products were sequenced and displayed a 100% sequence identity to mouse Src and Yes sequences in the NCBI database.

### Localization of SRC and Yes Protein throughout Preimplantation Development

SRC protein was detected at the 2-cell stage, by immunofluorescence, as a fluorescent ring encircling the periphery of each blastomere (Figure 1B). Upon reaching the 8-cell stage, SRC protein fluorescence diminished but reappeared in each blastomere of 8-cell compacting embryos, especially at areas of cellular contact in the basolateral domain of the plasma membrane. By the blastocyst stage, the cortical fluorescent pattern encircling each blastomere was maintained in both trophectoderm and inner cell mass (Figure 1B).

YES protein was also detected throughout mouse preimplantation development. At the 2-cell stage, the protein was diffusely localized in each blastomere, but the cortical distribution observed for SRC was also evident for YES (Figure 1B). The YES fluorescent signal also decreased at the 8-cell stage and became more obvious post compaction. The blastocyst displayed YES protein fluorescence in both ICM and TE cells, however YES protein fluorescence was never as intense as that observed for SRC protein in any preimplantation developmental stage (Figure 1B).

### Effects of Ouabain Treatment on SRC Activation

**Figure 2:**
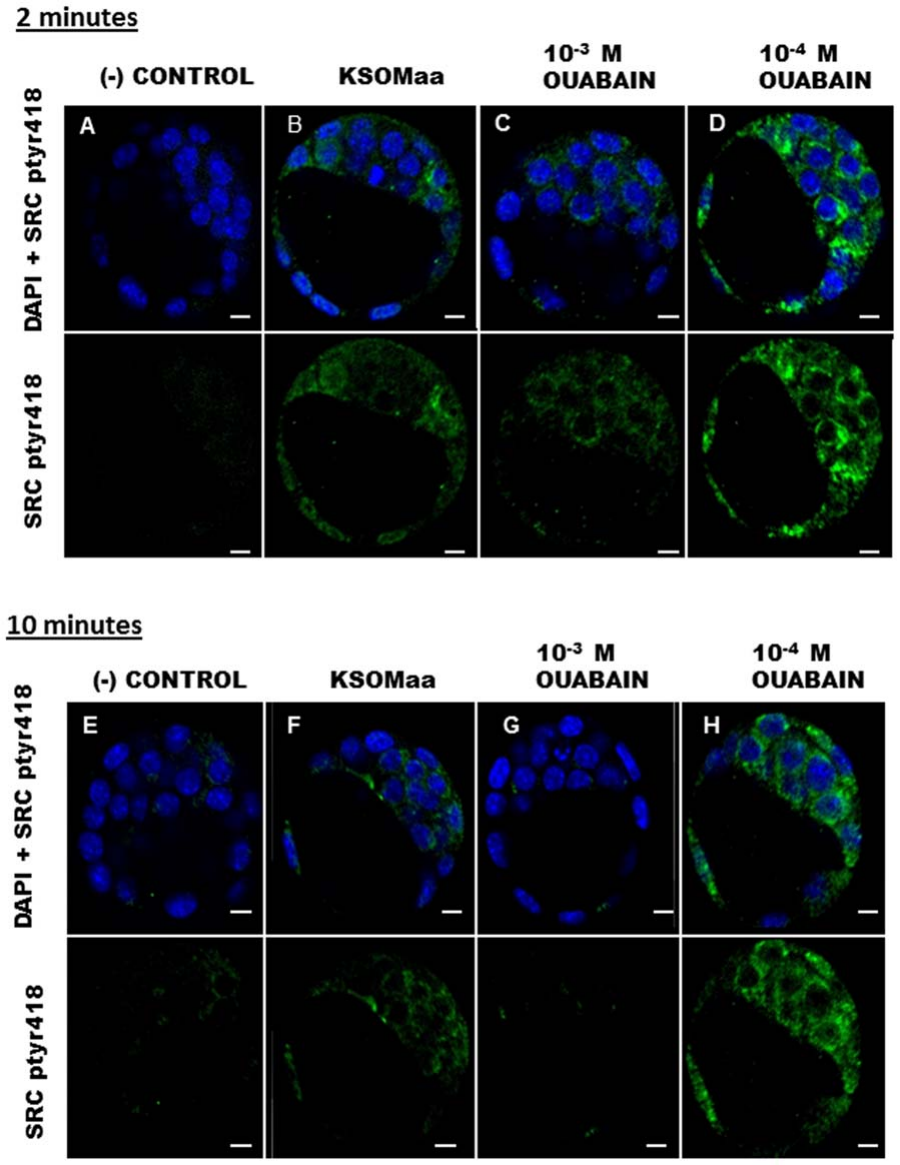
A–H. Effect of 2-minute and 10-minute Ouabain Treatment on SFK Phosphorylation and Activation at the Blastocyst Stage. Negative controls represent blastocysts exposed to FITC-conjugated secondary antibody only (no primary antibody) in addition to DAPI (**A,E**). The top row illustrates embryos merged with DAPI and FITC staining, while the bottom row illustrates FITC staining alone. In KSOMaa cultured controls, phosphorylated tyr418 of SRC was detected in the cytoplasm of blastocysts (**B,F**). Following a 2 minute treatment, phosphorylation decreased with (**C**) 10^−3^ M ouabain treated embryos but increased in (**D**) 10^−4^ M ouabain treated embryos. Following a 10 min treatment, phosphorylation fluorescence remained low (**G**) in 10^−3^ M ouabain treated embryos but remained high (**H**) in 10^−4^ M ouabain treated embryos. Green FITC staining indicates positive staining for phosphorylation of tyr418 on SRC and blue DAPI staining indicates nuclei. Scale bars are 10 µm.

We next investigated the effects of ouabain treatment on SFK activation during blastocyst formation. We employed standardized concentrations of ouabain (10^−3^ M), that are known to block the ion transport functions of the Na^+^/K^+^-ATPase in the mouse [14], [19], [23], [51]–[53] and concentrations (10^−4^ M) that do not. After a 2 minute ouabain treatment, blastocysts exhibited consistent variations in SRC tyr418 phosphorylation (Figure 2A). The 10^−3^ M ouabain concentration resulted in a marked reduction in tyr418 phosphorylation fluorescence, while treatment with 10^−4^ M ouabain for 2 minutes increased tyr418 phosphorylation fluorescence (Figure 2A). Both tyr418 phosphorylation fluorescence levels were compared to baseline levels in control embryos cultured in KSOMaa medium without drug and to no primary antibody controls. The total number of embryos used for no antibody (negative) control, KSOMaa, 10^−3^ M ouabain, and 10^−4^ M ouabain treatment groups, respectively, was 23, 21, 18, 26. These results demonstrate that ouabain treatment affects SRC phosphorylation at the blastocyst stage.

Following a 10 minute ouabain treatment, a greater difference in SRC tyr418 fluorescence in blastocysts was observed between treatment groups. Blastocysts cultured in 10^−3^ M ouabain displayed a prominent reduction in SRC tyr418 phosphorylation fluorescence when compared to KSOMaa controls (Figure 2B). In turn, blastocysts treated with 10^−4^ M ouabain for 10 mins exhibited increased SRC tyr418 phosphorylation fluorescence. The total number of embryos used for no antibody (negative) control, KSOMaa, 10^−3^ M ouabain, and 10^−4^ M ouabain treatment groups, respectively, were 10, 24, 32, 32. These results demonstrate the ability of different concentrations of ouabain and varying treatment times to induce disparate changes in SRC phosphorylation fluorescence in blastocysts. A high ouabain concentration, that blocks progression to the blastocyst stage, elicited reduced phosphorylation fluorescence levels on tyr418, indicating reduced SRC activity. A lower ouabain concentration that does not reduce development to the blastocyst stage elevated tyr418 phosphorylation fluorescence levels, indicating increased SRC activity.

### Effect of PP2 Treatment on Cavitation and SRC Phosphorylation

**Figure 3:**
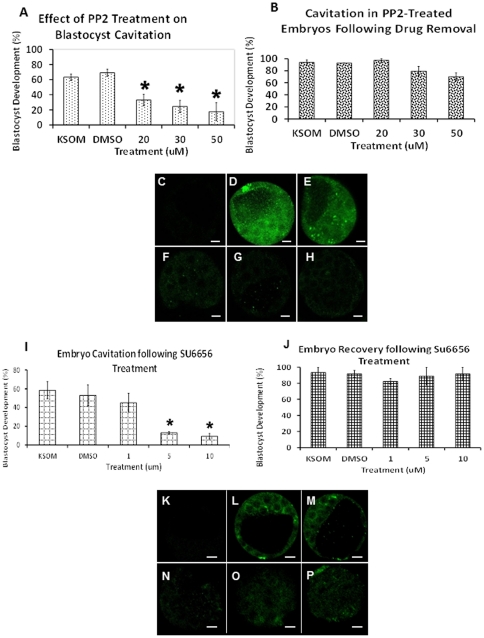
A–P. Effect of PP2 and SU6656 Treatment on Blastocyst Formation. Percentage of embryos developing from compacted morula to the blastocyst stage after 18 hours of culture in either KSOMaa, KSOMaa+DMSO, or KSOMaa plus 20 µM PP2, 30 µM PP2 or 50 µM PP2 (**A–H**). All PP2 concentrations significantly blocked the cavitation of morulae compared to KSOMaa or KSOMaa+DMSO vehicle controls (**A**). Standard error means are indicated by error bars. Percentage of embryos developing to the blastocyst stage after 18 hours of culture in either KSOMaa, KSOMaa+DMSO, and KSOMaa plus 20 µM PP2, 30 µM PP2 or 50 µM PP2 followed by a recovery culture period of 18 hours in KSOMaa (**B**). Embryos resumed development and reached the blastocyst stage, regardless of PP2 treatment, to levels comparable to either control group. (**B**). Whole-mount immunofluorescence labelling of blastocysts with anti-phospho-tyr418 of SRC (**C–H**). Negative control embryos that were incubated in FITC-conjugated secondary only (no primary antibody) (**C**). Images demonstrate baseline phosphorylation in (**D**) KSOMaa or (**E**) KSOMaa+DMSO controls and reduced phosphorylation after treatment with (**F**) 20 µM, (**G**) 30 µM, or (**H**) 50 µM PP2 for 18 hours. Percentage of morula cavitating after 18 hours of culture in KSOMaa, KSOMaa+DMSO, 1 µM SU6656, 5 µM SU6656 or 10 µM SU6656 (**I**). Experimental concentrations of 5 µM and 10 µM SU6656 significantly blocked cavitation when compared to KSOMaa control or KSOMaa+DMSO vehicle controls. Error bars indicate standard error means. Percentage of embryos developing to the blastocyst stage after 18 hours of culture in KSOMaa, KSOMaa+DMSO, 1 µM SU6656, 5 µM SU6656 or 10 µM SU6656 followed by a recovery culture period of 18 hours in KSOMaa (**J**). Treated embryos progressed to the blastocyst stage, regardless of SU6656 concentration, to levels comparable to either control treatment group. Whole-mount immunofluorescence labelling of blastocysts with anti phospho-tyr418 of SRC (**K–P**). Negative control embryos were exposed to FITC-conjugated secondary only (no primary antibody) (**K**). Images demonstrate baseline phosphorylation levels after treatment with (**L**) KSOMaa or (**M**) KSOMaa plus DMSO for 18 hours, and reduced phosphorylation after treatment with (**N**) 1 µM, (**O**) 5 µM, or (**P**) 10 µM SU6656 for 18 hours. Green FITC fluorescence indicates phosphorylation of tyr418 of SRC. Scale bars represent 10 µm.

Next we investigated the role of SFK signaling in regulating preimplantation development and more specifically on the regulation of trophectoderm tight junction permeability. Morulae treated with PP2 displayed a significant reduction in blastocyst formation after an 18 hour treatment at 20 µM, 30 µM and 50 µM, when compared to both control groups, P≤0.05 (Figure 3A). Of the control embryos, 63% of morulae cultured in KSOMaa cavitated, while 69% of DMSO+KSOMaa cultured embryos cavitated. For morulae cultured in 20 µM, 30 µM and 50 µM PP2, 33%, 25% and 18% of the treated embryos cavitated, respectively (Figure 3A). All embryos blocked from cavitating due to SFK inhibition were able to recover and progress to the blastocyst stage at levels comparable to control embryos when placed back into drug free KSOMaa media, P≤0.05 (Figure 3B). The respective cavitation frequencies for each recovered treatment group, presented with standard error of means are: 94±4%, 93±5%, 97±8%, 80%±8, 70±12% with the total number of embryos examined in each treatment group equaling 59, 78, 79, 70, 70. This result demonstrates that SRC activity facilitates blastocyst formation.

Embryos were randomly selected and removed from each treatment group (KSOMaa, DMSO+KSOMaa, 20 uM PP2, 30 uM PP2 and 50 uM PP2) following the initial 18 hour culture period and were fixed instead of being placed into the recovery KSOMaa culture experiment. Application of immunofluorescence methods for phosphorylation on tyr418 of SRC revealed a concentration dependent loss of tyr418 phosphorylation of SRC after treatment in PP2, when compared to embryos in the KSOMaa or DMSO+KSOMaa (Figure 3C–H). This outcome validated that PP2 treatment effectively blocks SRC phosphorylation on tyr418. The total number of embryos utilized for immunofluorescence for each treatment group was, respectively, 4, 8, 12, 15, 8, 12.

### Effect of SU6656 Treatment on Cavitation and SRC Activation

A second SFK inhibitor, SU6656, was used to test for consistency of outcomes after SFK inhibition. Morulae cultured in SU6656 exhibited a significant reduction in their ability to cavitate after an 18 hour treatment period, P≤0.05 (Figure 3I). Seventy-seven±7% of the embryos cultured under drug free KSOMaa conditions cavitated, while 63±8% of those cultured in DMSO+KSOMaa cavitated. Sixty-five±16% of the embryos cultured in 1 µM SU6656 cavitated while 18±5% in 5 µM treatment and only 16±6% of those in the 10 µM treatment cavitated. The total number of embryos in each group were, 40, 61, 47, 44, 43 respectively (Figure 3I). Once again the effects of treatment were reversible as blocked embryos placed into fresh KSOMaa after SU6656 treatment, recovered and cavitated to levels comparable to embryos cultured under control conditions, which were for the respective groups: 93±7%, 92±4%, 82±3%, 89±11% and 92±8% with the total number of embryos examined for recovery in each treatment group equaling 7, 26, 28, 24, 19. (Figure 3J) Embryos were selected and randomly allocated from the SU6656 treatment experiment from each treatment and fixed for use in indirect immunofluoresence. Embryos were tested for phosphorylation on tyr418 of SRC, which was blocked in a concentration dependent manner by SU6656 treatment when compared to either KSOMaa or DMSO+KSOMaa control groups (Figure 3K–P). The total number of embryos examined in no primary antibody (negative) control, KSOMaa, DMSO, 1 µM, 5 µM, or 10 µM SU6656 treatment groups was 3, 15, 20, 12, 12, 8. This result validates the efficacy of SU6656 to block phosphorylation of tyr418 in mouse preimplantation embryos. These results demonstrated the ability of a second SFK inhibitor to produce consistent outcomes. In total the results demonstrate that SFK activation is required for blastocyst formation to occur.

### Permeability of Trophectoderm Tight Junctions to 4 kDa FITC-Dextran Following Ouabain and PP2 Treatment

**Figure 4:**
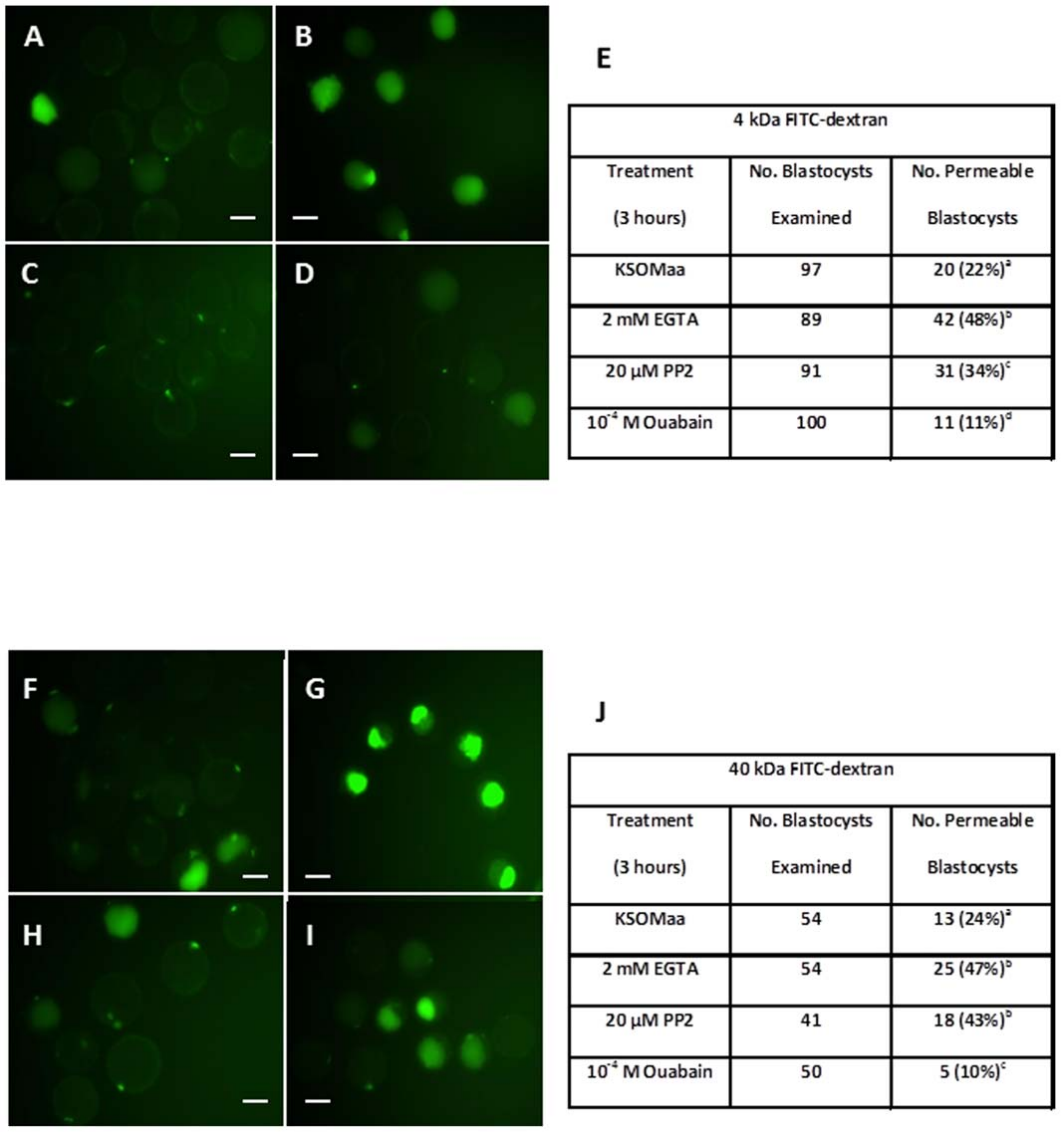
A–J. Effect of Ouabain and PP2 Treatment on Tight Junction Permeability using 4 kDa and 40 kda FITC-dextran in Blastocysts. Representative images of embryos following 3 hour treatment of (**A**) KSOMaa, (**B**) 2 mM EGTA, (**C**) 10^−4^ M ouabain, or (**D**) 20 µM PP2 using 4 kDa FITC-dextran. A significant difference was observed, P≤0.05, in the number of embryos displaying 4 kDa FITC-dextran fluorescence within the blastocyst cavity (**E**). Representative images of embryos following 3 hour treatment of (**F**) KSOMaa, (**G**) 2 mM EGTA, (**H**) 10^−4^ M ouabain, or (**I**) 20 µM PP2 using 40 kDa FITC dextran. A significant difference was observed, P≤0.05, in the number of embryos displaying 40 kDa FITC-dextran fluorescence within the blastocyst cavity, except between EGTA and PP2 treatment groups (**J**). The scale bars represent 100 µm.

4 kDa FITC-dextran was used to test the permeability of trophectoderm tight junctions in fresh flushed mouse blastocysts [23]. Of the blastocysts in KSOMaa for 3 hours (controls), 22±2% were permeable to 4 kDa FITC-dextran, showing the baseline permeability level of mouse blastocysts (Figure 4A,E). Blastocysts treated in 2 mM EGTA, which increased tight junction permeability, displayed 48±5% permeability to 4 kDa FITC-dextran (Figure 4B,E). Inhibiting SRC through treatment with 20 µM PP2 resulted in 34±1% of the blastocysts displaying permeablity to 4 kDa FITC-dextran, while treating with 10^−4^ M ouabain decreased tight junction permeability to 11±3% (Figure 4C–D,E). The total number of embryos placed in each group were 97, 89, 91, 100 (Figure 4E). All treatment groups were statistically significant to each other when analyzed using the Student-Neuman-Keul analysis method, P≤0.05. These results suggest a relationship between SRC activity and tight junction function in the mouse blastocyst, since SRC inhibition increased tight junction permeability and SRC activation decreased tight junction permeability.

### Permeability of Trophectoderm Tight Junctions to 40 kDa FITC-dextran Following Ouabain and PP2 Treatment

A second, larger, molecular weight FITC-dextran was utilized to further demonstrate the effects on tight junction permeability in blastocysts after treatments identical to the 4 kDa FITC-dextran trial. These experiments demonstrated baseline permeability of blastocysts to 40 kDa FITC-dextran was 24±2% (Figure 4F,J). Embryos treated with 2 mM EGTA displayed an increased permeability to 40 kDa FITC-dextran with 48±3% of treated blastocysts accumulating FITC-dextran in their cavities (Figure 4G,J). Those blastocysts treated in 20 µM PP2 were comparable to the EGTA treated group with 43±2% of permeable blastocysts, while treatment with 10^−4^ M ouabain reduced tight junction permeability resulting in 10±2% of treated blastocysts accumulating FITC-dextran (Figure 4H,I,J). The number of embryos examined for each respective treatment group were: 54, 54, 41, 50 (Figure 4J). Analysis of this data set using the Student-Neuman-Keul method demonstrated statistically significant differences among all groups, except between EGTA and 20 µM PP2 treated blastocysts, P≤0.05. The overall conclusion is that PP2 treatment increased tight junction permeability equally well as EGTA treatment. Thus SRC activity is a potent regulator of tight junction permeability during mouse blastocyst formation.

## Discussion

Our outcomes demonstrate that mRNAs encoding SFK family members SRC and YES are detectable during preimplantation development including blastocyst stage mouse embryos. Ouabain treatment has concentration dependent effects on SFK activation; higher concentrations known to block Na^+^/K^+^-ATPase transport activity in mouse embryos reduce SRC activation, and lower ouabain concentrations that do not block embryonic Na^+^/K^+^-ATPase ion transport enhance SRC activation. Blockade of SFK activation reduces blastocyst cavitation in a concentration dependent and in all cases a reversible manner, and more importantly, increases trophectoderm tight junction permeability. Conversely, ouabain treatment (10^−4^ M) that increases SFK phosphorylation decreases trophectoderm tight junction permeability. In total our results demonstrate that SFK activity facilitates blastocyst formation and that SFKs are a potent mediator of trophectoderm tight junction function in the blastocyst.

We first investigated the presence of SFK mRNAs during preimplantaiton development to provide a foundation for our studies. Our main focus was to determine if Src and Yes mRNAs were present prior to and following blastocyst formation. There are eight known SFK family members, *Src* and *Yes*, along with *Fyn* are ubiquitously expressed [54]. *Fgr* is present in B cells and myeloid cells, as are *Lyn*, *Lck*, *Blk* and *Hck*, which are also present in T cells and brain tissue [54]. This more restricted expression pattern would suggest that only *Src*, *Yes* and *Fyn* would be expected to be present during early mouse development. We restricted our analysis to include only Src and Yes as we were only able to obtain reliable antisera for these two members of the SFK family. The detection of mRNAs for Src and Yes during preimplantation development and most importantly within blastocysts implies a role for these SFK family members in regulating early development and allowed for experiments aimed at determining which kinases could be tethered to the Na^+^/K^+^-ATPase pump and activated in the signaling pathway regulating tight junctions in the preimplantation embryo. However, our results do not validate that only *Src*, and *Yes* have a role to play in blastocyst formation as it is quite possible proteins from other family members may be present in the blastocyst and contribute to the outcomes reported in our study. It will be important in the future to obtain effective antisera for these additional family members to characterize their localization patterns during preimplantation development.

Immunofluorescence revealed SRC and YES protein localization patterns also during preimplantation development and most importantly at the morula and blastocyst stages. SRC fluorescence was more intense when compared to YES, however our results do not precisely measure the abundance of these proteins. Immunofluoresence is a sensitive method that is very effective at revealing protein localization patterns. While several studies have developed ways to quantify immunofluoresence signals it is challenging to standardize conditions sufficiently to allow for a precise quantitiation of target proteins levels to occur. Because of this limitation we did not attempt to precisely measure variations in immunofluorescence signals in this study. We have confined ourselves to simply reporting consistent localization patterns and in providing qualitative assessment of consistent and obvious changes in fluorescence intensity following ouabain treatment. This is a limitation of our study but providing quantitative fold differences in fluorescence intensity may be misleading as its precise relationship to protein abundance or phosphorylation cannot be assured.

SRC has been linked to ouabain induced Na^+^/K^+^-ATPase signalling in other cell systems such as cardiac myocytes, and speculation exists that the other SFKs could be a part of their own signalling pathway specific to different tissues [46], [47]. The pattern of protein localization was consistent for SRC, which was detectable at cell margins within the 2-cell embryo, diminishing during compaction, reappearing afterwards and present in all cells of the blastocyst. The significance of appearing at basolateral cell domains cannot be overlooked, as this pattern mimics the well-known distribution of the Na^+^/K^+^-ATPase in the trophectoderm [19], [22], [25], [55], [56]. Therefore SRC, and possibly YES, may be co-localized with the Na^+^/K^+^-ATPase pump in a signalling complex regulating tight junctions. Although it is possible that all SFKs interact with the Na^+^/K^+^-ATPase because of their similar structure, it would likely be in a tissue specific manner due to the difference in signals and protein distribution among cell types [46], [47]. Another intriguing possibility is that the activation of a specific SFK may elicit specific Na^+^/K^+^-ATPase responses.

Many studies report that high concentrations of ouabain will block the ion transport function of the rodent Na^+^/K^+^-ATPase α1-subunit, while lower concentrations will activate signalling pathways transduced through SRC [20], [23], [32], [34], [38], [40], [47]–[49], [51], [53]. Low CTS concentrations are demonstrated to stimulate Na^+^/K^+^-ATPase activity in cardiac myocytes, by way of activating SRC/EGFR, PI3K and ROS production [44], [48], [49]. Na^+^/K^+^-ATPase α1 knockout studies have even demonstrated increased basal SRC activity, and the loss of ouabain induction of SRC signalling (reviewed in [40]–[42]. In other studies, using SYF (*Src*, *Yes*, *Fyn* triple deletion) cells or pre-treating cells with PP2, caused ouabain to lose its ability to activate ERK [44], [46]–[49].

Studies report maximal ouabain effects between 2 and 5 minutes, followed by depletion of its effects in cells [57], [58]. Our study suggests ouabain regulation of SRC activity occurs through their mutual association with the Na^+^/K^+^-ATPase pump. Although these outcomes were consistent and novel, the results did not convincingly exhibit a maximal effect following 2 minutes. Following 10 minutes of ouabain treatment, obvious differences between the level of fluorescence of tyr418 was observed between treatment groups. Blastocysts treated with 10^−3^ M ouabain exhibited a reduction in tyr418 phosphorylation fluorescence while those treated in 10^−4^ M ouabain demonstrated an increase in tyr418 phosphorylation fluorescence. Of greater significance, the percentage of embryos exhibiting higher tyr418 phosphorylation fluorescence doubled in the 10 minute group when compared to blastocysts treated for 2 min. The 10 minute treatment also resulted in higher intensity fluorescence when compared to the 2 min group, suggesting a time dependent aspect of ouabain stimulated SRC activity. This outcome has revealed a novel mode of SRC regulation in the blastocyst. This, together with the localization of SRC protein to the basolateral domain of embryonic cells, and the existing literature showing ouabain eliciting its effects through the Na^+^/K^+^-ATPase, implicates the pump in this signalling pathway in the mouse blastocyst. An important limitation of our study is that we were not able to precisely measure these changes in tyr418 phosphorylation by employing methods such as Western blotting. The primary reason for this was attempts to perform this analysis using samples derived from several 100 blastocysts per lane failed in detecting either total SRC, Yes or phosphorylated SRC. This is not completely surprising as the application of Western blotting methods to quantify protein changes during preimplantation stage embryos often fail even with the application to samples derived from 100's of early embryos due to low overall protein abundance in such samples. While we do not report specific fold changes in fluorescence the observations we do report were consistent and while qualitative in nature due reflect obvious changes in fluorescence intensity representative of tyr418 phosphorylation. We have validated the specificity of the antisera used in this study by conducting tissue and cell sample Western blots (Figure S1). In addition we employed commercially sourced antisera that have been employed and validated in several publications employing them for the same purpose we have in this study [59]–[62].

PP2 is a widely used specific and effective SFK inhibitor [63]. PP2 binds between the two lobes of the kinase domain and prevents usual SRC substrates from binding [64]. PP2 always binds near the ATP binding pocket, and since the sequences adjacent to the ATP binding pockets are very different between the SFKs, these areas may be the true binding sites [64]. PP2 concentrations used were 20, 30 and 50 µM and were compared to culture control KSOMaa and vehicle control KSOMaa plus DMSO. The first set of experiments demonstrated all concentrations of PP2 significantly blocked morulae from developing into blastocysts when compared to either control group. Importantly, the majority of embryos were able to recover from the treatment and reach the blastocyst stage once thoroughly washed. Immunofluorescence demonstrated that PP2 blocked phosphorylation of tyr418 of SRC. These results together demonstrate the significance of SFK activity for blastocyst development.

Further confirmation of these outcomes was provided by culturing morulae in 1, 5 or 10 µM of SU6656, which produced identical results to the PP2 experiments. SU6656 selectively inhibits SRC, YES and FYN, although SRC is 6.5 fold more sensitive than the other two SFKs [65]. It acts by competitively competing for ATP but does not affect MAPK activation [65]. SU6656 is highly specific and has greater potency of action than PP2 and does not block any other molecule to the degree that it inhibits SRC [65]. The two higher doses of SU6656 used significantly blocked morulae from becoming blastocysts. The concentrations of SU6656 used in this study are well defined [66], those of PP2 are in the micromolar range in order to elicit its effect on intact cells, such as embryos, as reported in literature [67]. Our studies demonstrated no difference in effects on embryonic cavitation with 1 µM, 5 µm, or 10 µM PP2. An additional limitation of our study is that while we determined that SRC inhibition treatments were reversible and treated embryos were able to proceed to the blastocyst stage once removed from treatment, we did not measure cell numbers in the recovered blastocysts. The primary reason for this was that we were focused on ensuring that our treatment levels were not embryo toxic and that an ability to reach the blastocyst stage following treatment certainly indicates that the concentrations used were not embryo toxic. However our results do not in any way suggest that embryo viability was not compromised as we did not test for this, which would require embryo transfer experiments and assessment of pregnancy outcomes which was beyond the scope of the present study.

Tight junctions between adjacent cells form a continuous barrier to epithelial paracellular transport [12], [28], [29], [68], [69]. They provide the impermeable seal allowing fluid accumulation within the blastocyst cavity and contribute to the polarized distribution of the Na^+^/K^+^-ATPase pump in the basolateral membrane of the trophectoderm [12], [28], [29], [68], [69]. Tight junctions play an integral role in blastocyst development and as such, our primary objective was to investigate whether SRC activity regulates trophectoderm tight junction function during blastocyst development. Although Wiley [53] reported that high or low doses of ouabain had no effect on the appearance of tight junctions, the effects of ouabain treatment on tight junction function were not investigated in that study.

The FITC-dextran permeability assay is a sensitive method ideally suited for assessing tight junction functionality [23], [31]. By visualizing the amount of the molecule permeating the tight junctions and accumulating within the blastocyst cavity, conclusions can be drawn regarding their permeability and thus function. In these experiments, we observed that inhibition of SFK by PP2 increased tight junction permeability to a percentage comparable to the positive control EGTA. Of the approximately 100 embryos examined in each treatment group incubated in 4 kDa FITC-dextran, 48% were permeable when treated in EGTA, while 34% were when treated with PP2. These percentages were significantly different from each other signifying that PP2 was not as effective as EGTA in opening tight junctions. However, the PP2 treated embryos were more permeable to 4 kDa FITC-dextran when compared to KSOMaa control treated embryos, which exhibited 22% permeability.

This effect was also observed using 40 kDa FITC-detxtran, which showed no statistical significance between the EGTA and PP2 treated groups. This outcome establishes that SFK inhibition is just as effective at disrupting tight junction function as EGTA treatment. Remarkably, 10^−4^ M ouabain affected tight junction function by decreasing their permeability to 4 or 40 kDa FITC-dextran. Both experimental outcomes demonstrate that SRC activation is associated with decreased trophectoderm tight junction permeability. Recently, it has been suggested that SRC may be able to directly or indirectly interact with ZO-1 and this provides a route for understanding how SRC activation may regulate tight junction function [70]. Our earlier studies (Violette et al., [23] established that high ouabain treatment (10^−3^ M) is associated with disrupted tight junction associated protein (ZO-1 and occluden) distribution in treated blastocysts. In addition, it was reported by Violette [23] that high ouabain treatment increased tight junction permeability. These outcomes can now be understood due to our present study, as our findings indicate that high ouabain treatment would reduce SRC activation and this in turn would result in increased trophectoderm tight junction permeability. Recently, we also discovered that down-regulation of the Na^+^/K^+^-ATPase β1-subunit results in a loss of blastocyst formation, also associated with a disrupted trophectoderm tight junction associated protein distribution [22]. It is thus intriguing to consider the possibility that the Na^+/^K^+^-ATPase β1-subunit may also be subject to SFK regulation. These will represent the subject of our future experiments. In addition, the ouabain-Na^+^/K^+^-ATPase-SRC complex is often associated with activation of ERK1/2, and now that SRC is implicated in interacting with ZO-1, regulation of trophectoderm tight junctions through this signalling cascade is very likely.

In conclusion, this study has investigated the unconventional function of the Na^+^/K^+^-ATPase pump as a signal transducer, rather than an ion transporter responsible for maintaining osmotic gradients. A number of novel findings pertaining to blastocyst development have been uncovered. Firstly, *Src* and *Yes* mRNAs and protein are detectable at the blastocyst stage. Secondly, 10^−3^ M ouabain reduces SFK activity, whereas 10^−4^ M ouabain increases tyr418 phosphorylation. Localizing SRC in the basolateral domain of blastocyst cells, and identifying that SFK activity is regulated by CTS, strongly suggests the involvement of the Na^+^/K^+^-ATPase in transducing this signal. Thirdly, SFK activity is required for blastocyst development. Lastly, SFKs regulates trophectoderm tight junction function in the blastocyst. Considering that little is known of how tight junctions could be regulated in the blastocyst, or of the signaling cascade initiated by CTS binding to the Na^+^/K^+^-ATPase in embryos, this study has increased our understanding of the mechanisms controlling blastocyst formation. It would seem to be reasonable to propose that the Na^+^/K^+^-ATPase is present in caveolae of the trophectoderm, creating a signalosome capable of transducing SFK signals following ouabain activation of the Na/K-ATPase. This research therefore addresses the basic mechanisms controlling early mammalian development and has uncovered new principles for understanding how blastocyst formation is regulated.

## Materials and Methods

### Mouse Preimplantation Embryo Collection

Female CD1 (3–5 weeks of age; The Jackson Laboratory, Bar Harbor, Maine, USA) or MF1 mice (3–5 weeks of age; Harlan Sprague Dawley, Indianapolis, IN, USA) were superovulated by intraperitoneal (IP) injection of 5 IU pregnant mare's serum gonadotropin (PMSG, Intervet Canada Ltd, Whitby, ON, Canada) followed 48 hours later by 5 IU human chorionic gonadotropin (hCG; Intervet Canada Lts., Whitby, ON, Canada). Superovulated female mice were mated with CD1 males (8–52 weeks of age, Charles River, Quebec, Canada). Successful mating was indicated by the presence of a vaginal plug the following morning (day 1). Time post-hCG was used to measure the developmental age of the embryos. Preimplantation mouse embryos were collected at 18 hours (1-cell), 48 hours (2-cell), 55 hours (4-cell), 66 hours (8-cell), 77 hours (morula) and 90 hours (blastocyst) post hCG. All embryo stages were flushed from the reproductive tract using M2 medium (Sigma, St.Louis, MO, USA). Embryos were washed several times in M2 medium with HEPES or KSOMaa and collected in pools to be 1) frozen at −80°C until their use in RNA extraction; 2) fixed for indirect immunofluorescence; or 3) cultured in EmbryoMax® KSOMaa (potassium simplex optimized medium with amino acids) Liquid Mouse Embryo Media (Chemicon International – Specialty Media, Temecula, CA, USA) under mineral oil and maintained in culture under 5% CO_2_ in air atmosphere at 37°C for the time outlined by the experimental design. All animal care and embryo collection methods were conducted using approved protocols from the Canadian Council of Animal Care and the University of Western Ontario Animal Care and Veterinary Services. The approved University of Western Ontario animal care protocol number for these studies is 2010-021Watson.

### RNA Extraction

Total RNA was extracted from pools of 20 or 40 preimplantation stage mouse embryos, stored at −80°C in M2 medium with HEPES immediately after collection, using the PicoPure® RNA Isolation Kit (Arcturus, Molecular Devices, Sunnyvale, CA, USA). Samples were treated with DNAse I (Qiagen Inc., Mississauga, ON, Canada) to remove any possible DNA contamination.

### Reverse Transcription and Polymerase Chain Reaction (RT-PCR)

Total RNA was reverse transcribed (RT) using the suggested protocol of Sensiscript RT (Qiagen Inc., Mississauga, ON, Canada). Each sample was diluted to a concentration of one embryo equivalent per microliter. PCR reactions were carried out in a volume of 50 µL consisting of 5 µL 10× PCR buffer, 2 µL 50 mM magnesium chloride, 1 µL 10 mM dNTP (all above from Invitrogen Life Technologies, Burlington, ON, Canada), 1 µM of each appropriate PCR primer, 38.8 µL HyPure™ water (Hyclone, Thermo Fisher Scientific Inc., Ottawa, ON, Canada), 0.2 µL Platimum® Taq DNA Polymerase (Invitrogen Life Technologies, Burlington, ON, Canada) and 1 µL cDNA for the embryonic stage being tested. Amplification reactions were performed using a Techne Touchgene Gradient DNA thermal cycler (Techne Inc., Burlington, NJ, USA). PCR reactions were repeated a minimum of three times using cDNA prepared from embryos at each indicated stage, isolated from a minimum of three developmental series (2-cell, 4-cell, 8-cell, morula and blastocyst stages). Positive (lung tissue cDNA) and negative control (no cDNA template) samples were included for each primer set in each experiment. Lung RNA was extracted by the Trizol Reagent® protocol suggested by the manufacturer (Molecular Research Center Inc., Cincinnati, OH, USA). The identity of each PCR product was confirmed by sequence analysis which resulted in a 100% sequence identity for both the Src and Yes PCR products derived from embryo and control samples (DNA Sequencing Facility, Robarts Research Institute, London, On, Canada).

### Primer Design

Primer pairs for PCR were designed and synthesized (Invitrogen Life Technologies, Burlington, ON, Canada) for *Src*, and *Yes*, based on available mouse nucleotide sequences in GenBank.

### Antisera

Rabbit polyclonal antiserum raised against the phosphorylated tyrosine residue 418 of human SRC (ab481650, abcam, Cambridge, MA, USA) was utilized at a dilution of 1∶100 to localize SRC phosphorylation in mouse embryos. Rabbit polyclonal antisera against total human SRC (#2108, Cell Signaling Technology, Danvers, MA, USA) and human YES (#2734, Cell Signaling Technology, Danvers, MA, USA) were used at dilutions of 1∶200 to localize the proteins in mouse embryos. Western blotting utilizing standard SDS-polyacrylamide Gel Electrophoresis (PAGE) and chemi-luminescence detection methods was applied to verify the specificity of the SRC and YES antisera employed in the study (Figure S1).

### Whole-mount Indirect Immunofluorescence and Confocal Microscopy

Mouse preimplantation stage embryos were collected from the reproductive tracts of superovulated female mice as described above. Embryo pools were fixed in 2% paraformaldehyde in PBS for 20 minutes at room temperature. Fixed embryos were incubated in embryo blocking buffer (0.01% Triton X-100 and 5% Normal Donkey Serum in 1× PBS) for 1 hour followed by one wash in 1× PBS for 20 minutes at 37°C. Embryos were incubated with primary antisera diluted in antibody dilution buffer (ADB) (0.005% Triton X-100 and 1% Normal Donkey Serum in 1× PBS) for 1 hour at 37°C or overnight at 4°C. Embryos were then washed 3× in ADB for 30 minutes at 37°C, then incubated with FITC-conjugated secondary antibody (Jackson ImmunoResearch Laboratories Inc., West Grove, PA, USA) at a 1∶200 dilution in ADB for 1 hour at 37°C or overnight at 4°C. To stain nuclear DNA, the first 30 minute wash in ADB included 4′,6-diamindino-2-phenylindole dihydrochloride (DAPI) (Sigma-Aldrich Cananda Ltd., Oakville, ON, Canada), diluted to 1∶2000 from 1 mg/mL stock solution. Two additional washes in 400 µL ADB of 30 minutes each at 37°C were applied. Fully processed embryos were mounted onto glass slides in a drop of Vectashield (Vector Laboratories; Burlington, Ontario, Canada) under Vaseline-elevated glass coverslips and sealed with nail polish. Immunofluorescence imaging employed an Olympus Fluoview 1000 laser scanning confocal microscope. For all immunofluorescence protocols, embryos serving as negative controls were incubated in ADB rather than primary antibody.

### Effects of Ouabain Treatment on Blastocyst SRC Activity

Culture drops of KSOMaa were equilibrated at 37°C and 5% CO_2_ in air atmosphere for 1 hour. Blastocysts were flushed from mouse uteri, washed thoroughly through M2 medium with HEPES, then KSOMaa and were incubated in 20 µL KSOMaa drops for 1 hour, after which they were placed in KSOMaa/ouabain (10^−3^ M, 10^−4^ M) for 2 or 10 minutes. Immediately following ouabain treatment, blastocysts were fixed in 2% paraformaldehyde for 20 minutes at room temperature and were then immunostained for phosphorylation of tyr418 of SRC.

### SFK Inhibition of Mouse Embryos (PP2, SU6656)

Culture drops of KSOMaa, DMSO (vehicle control for PP2), and PP2 (20 µM, 30 µM, 50 µM) were incubated for 1 hour at 37°C and 5% CO_2_ in air atmosphere. Morulae were flushed from uteri, washed thoroughly through M2 medium with HEPES, then KSOMaa and placed into 20 µL culture drops of KSOMaa, or KSOMaa plus DMSO, or KSOMaa plus PP2. Morulae were cultured for 18 hours at which time progression to the blastocyst stage was assessed. SU6656 at concentrations of 1 µM, 5 µM, 10 µM were also used in the same experimental design described above.

### Measurement of Tight Junction Function by FITC-Dextran Uptake Assay

To investigate the effects of SRC inhibition on tight junction permeability, blastocysts were flushed from uteri, were washed through KSOMaa and placed into 15 µL culture drops of KSOMaa, KSOMaa/2 mM EGTA (ethylene glycol Bis- (β-aminoethyl ether) N,N,N,N -tetraacetic acid), KSOMaa/10^−4^ M ouabain or KSOMaa/20 µM PP2 for 3 hours. After the 3 hour time point, blastocysts were transferred to 4 or 40 kDa FITC-dextran in KSOMaa for 30 minutes. Following this incubation, blastocysts were immediately washed in three 50 µL wash drops of KSOMaa and placed in a fourth clean KSOMaa drop for immediate visualization using a Zeiss fluorescent compound microscope equipped with epi-fluorescent optics.

### Statistical Analysis

Statistical analysis of data was performed using SigmaStat®, version 3.5, software. The results from culture in ouabain, PP2 and SU6656 are all presented as the mean ± standard error from three independent experimental replicates. For testing significant differences between culture groups, data were subjected to one-way analysis of variance (ANOVA). Data analysis for ouabain and PP2 trials passed normality tests and were followed by the Holm-Sidak post hoc test. Data for the SU6656 and tight junction permeability experiments were evaluated by applying an ANOVA on ranks. As a post-hoc test, the Student-Neuman-Keul method was employed. For all data analysis, P≤0.05 was considered statistically significant.

## Supporting Information

Figure S1 **A,B. Supplementary Data – Src and Yes antiserum western blotting in control tissues.** SRC and YES antibodies were tested on liver, pancreas, mammary and cancerous acinar cell line control protein extracts (**A,B**). The SRC antiserum resulted in the detection of a single band of expected molecular weight (60 kDa) in liver and AR42J (**A**). SRC protein was not detected in mammary protein samples but was detected in both total pancreas tissue samples (**A**). The doublet detected in pancreatic tissue is suggestive of SRC phophorylation in this tissue (**A**). Western blot conducted using the YES antiserum also validated the specificity of the antiserum in the two pancreas protein tissue samples by producing bands of expected size in each sample (**B**). Yes protein was undetectable in liver, mammary gland tissue or AR42J cells (**B**).
(TIF)
