# Supplementary figures and images for: Ouabain Stimulates a Na+/K+-ATPase-Mediated SFK-Activated Signalling Pathway That Regulates Tight Junction Function in the Mouse Blastocyst
Source: PLoS One. 2011 Aug 25;6(8):e23704. doi: 10.1371/journal.pone.0023704 (PMC3162003; doi:10.1371/journal.pone.0023704)

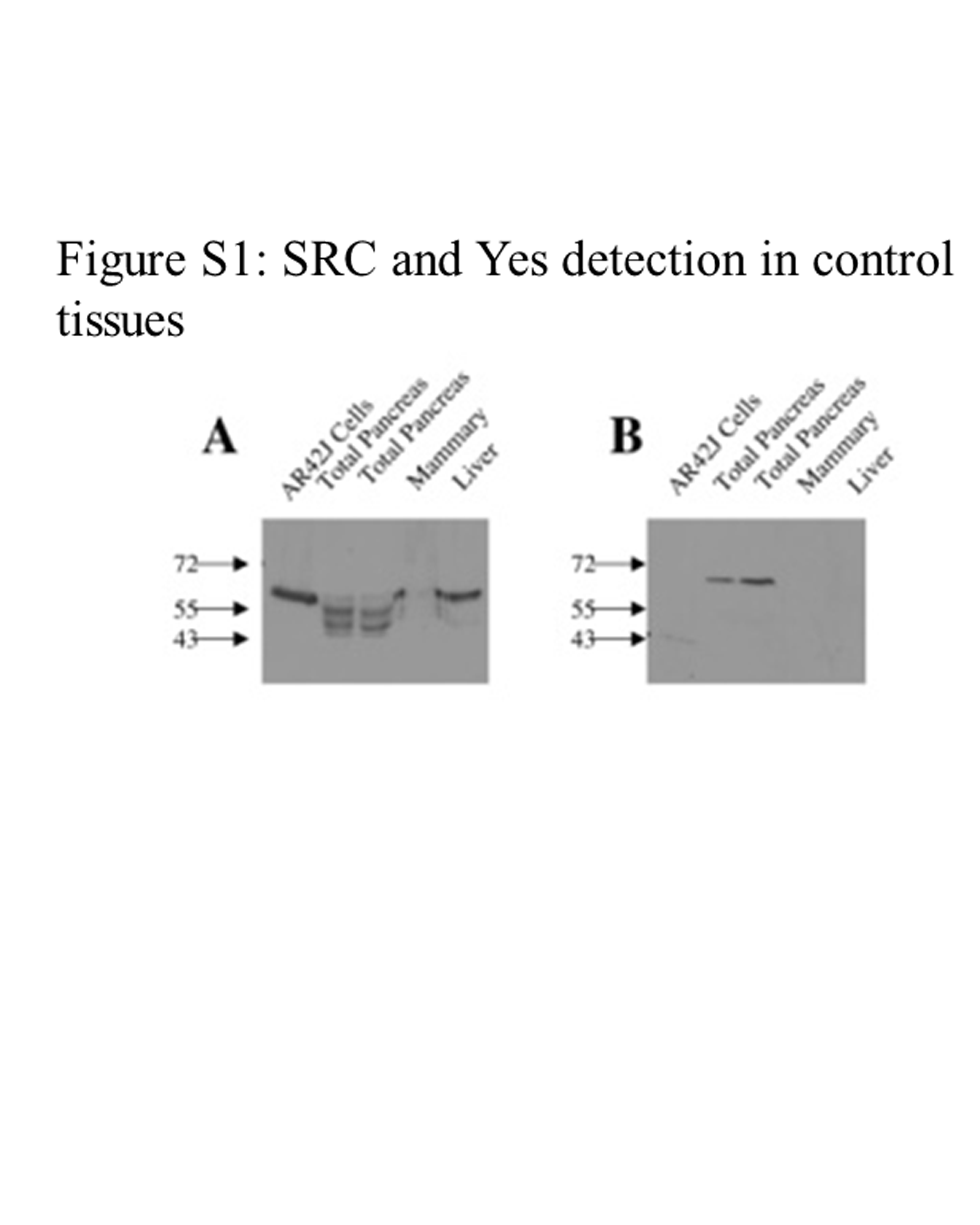

Supplement: Figure S1 — A,B. Supplementary Data – Src and Yes antiserum western blotting in control tissues. SRC and YES antibodies were tested on liver, pancreas, mammary and cancerous acinar cell line control protein extracts (A,B). The SRC antiserum resulted in the detection of a single band of expected molecular weight (60 kDa) in liver and AR42J (A). SRC protein was not detected in mammary protein samples but was detected in both total pancreas tissue samples (A). The doublet detected in pancreatic tissue is suggestive of SRC phophorylation in this tissue (A). Western blot conducted using the YES antiserum also validated the specificity of the antiserum in the two pancreas protein tissue samples by producing bands of expected size in each sample (B). Yes protein was undetectable in liver, mammary gland tissue or AR42J cells (B). (TIF) [file pone.0023704.s001.tif]
